# Supplementary material for: Long-Term Application of a Synbiotic Chitosan and Acinetobacter KU011TH Mixture on the Growth Performance, Health Status, and Disease Resistance of Hybrid Catfish (Clarias gariepinus × C. macrocephalus) during Winter
Source: Microorganisms. 2023 Jul 14;11(7):1807. doi: 10.3390/microorganisms11071807 (PMC10385702; doi:10.3390/microorganisms11071807)
Supplement: Supplementary file 1 [file microorganisms-11-01807-s001.zip › microorganisms-2377287-supplementary.pptx]

## Slide 1
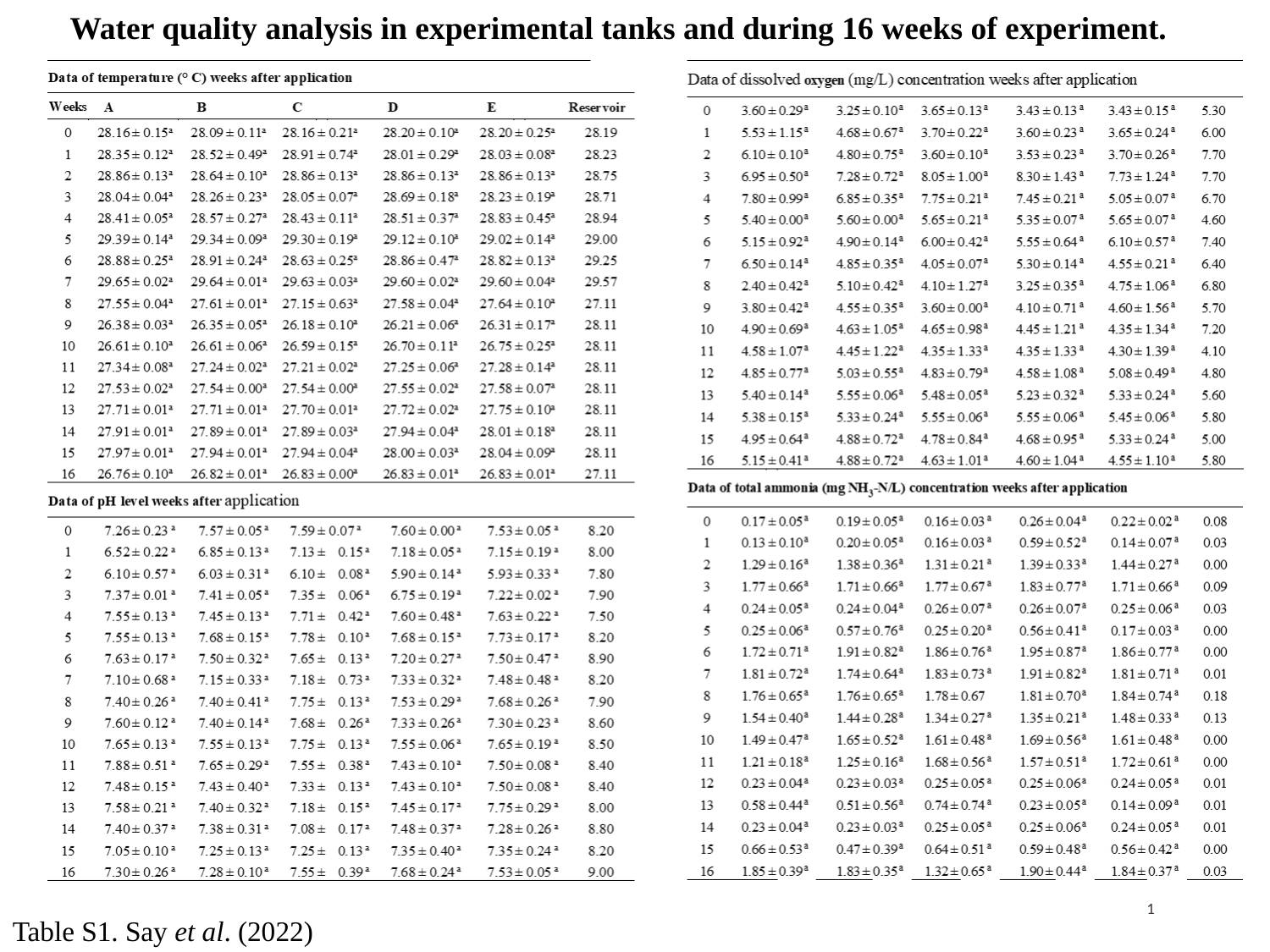

Water quality analysis in experimental tanks and during 16 weeks of experiment.
1
Table S1. Say et al. (2022)

## Slide 2
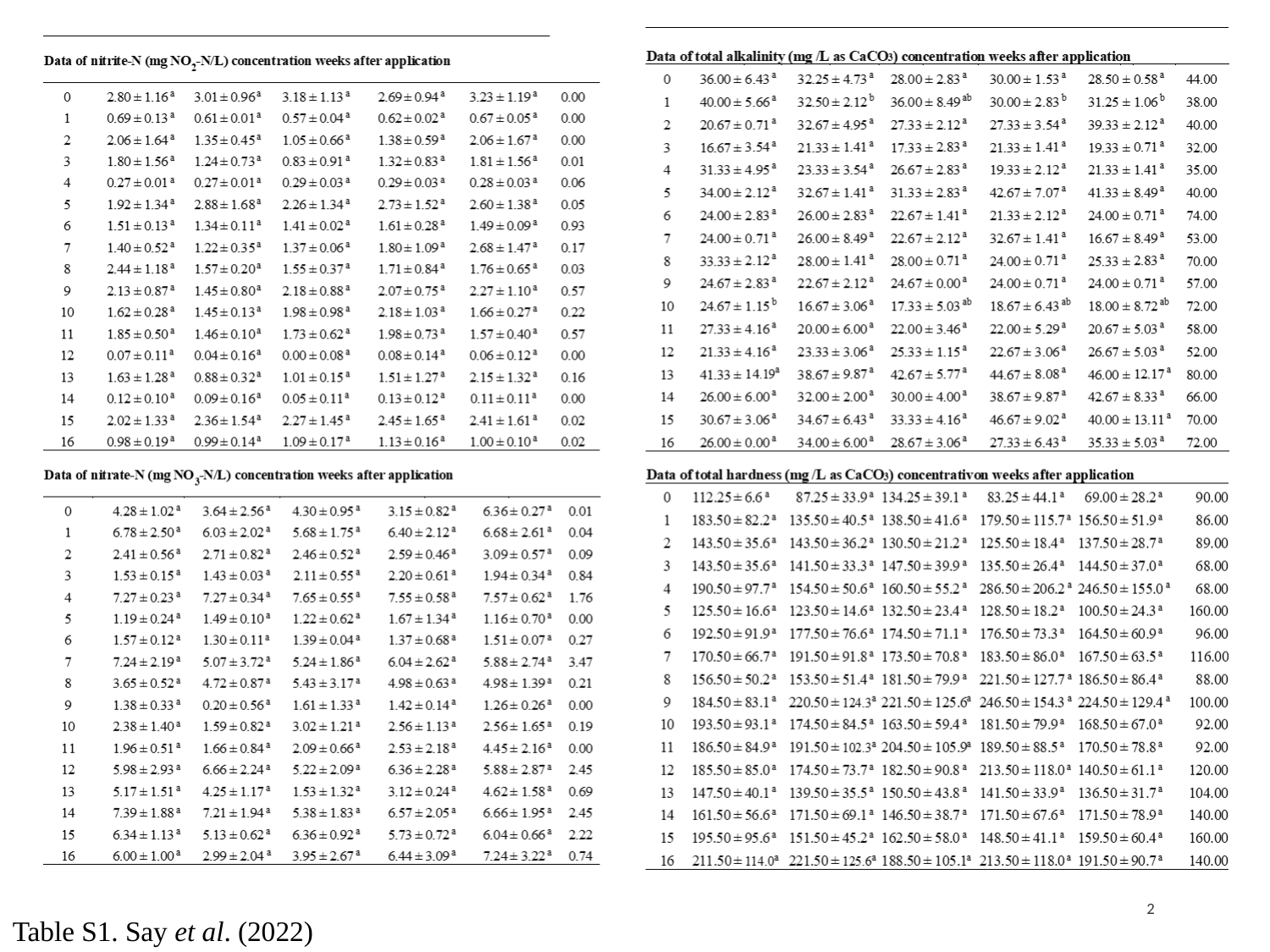

2
Table S1. Say et al. (2022)
